# Endoscopic Ultrasound-Guided Transrectal Pelvic Abscess Drainage (EUS-PAD) – Bridging to Surgery: Report of Two Cases

**DOI:** 10.1055/s-0043-1777791

**Published:** 2023-12-19

**Authors:** Tina Goerl, Christoph Speck, Alexander Gehring, Reiko Wiessner

**Affiliations:** 1 Klinik Für Allgemein- und Viszeralchirurgie, Bodden-kliniken Ribnitz-Damgarten, Ribnitz-Damgarten, Germany; 2 Klinik Für Innere Medizin II/Gastroenterologie, Bodden-kliniken Ribnitz-Damgarten, Ribnitz-Damgarten, Germany

**Keywords:** endoscopic ultrasound, pelvic abscess drainage, transrectal surgery, bridging to surgery

## Abstract

**Background** Currently, the methods for drainage of pelvic abscess primarily use computed tomography- or ultrasound-guided percutaneous drainage or surgical drainage. Endoscopic ultrasound-guided pelvic abscess drainage (EUS-PAD) is an alternative, minimally invasive tool to drain an abscess, localized close to the rectum or left colon, and therefore not accessible by other means.

**Methods** We report on the success of endoscopic ultrasound-guided drainage of peridiverticulitic abscess based on the two cases presented here. Using endoscopic ultrasound guidance an aspiration of the abscess from the endoluminal could be realized. After successive balloon dilatation via a guidewire while using X-ray imaging, the placement of pigtail or flap stent was performed. In addition, conservative therapy measures such as antibiotics, diet, and pain management were performed.

**Results** The interventions were successful in both patients, resulting in rapid recourse of discomfort, abscess size, and sepsis. After controlling the consequences of complicated diverticulitis, both patients underwent laparoscopic sigmoid resection with primary anastomosis and without ileostomy during an inflammation-free interval.

**Conclusion** Both cases demonstrate the advantages of EUS-PAD. A laparoscopic operation with primary anastomosis, lower perioperative risk, and without need of a protective ileostomy in early elective setting became possible by bridging the time until surgery by using EUS-PAD.

Pelvic abscesses result from diverticulitis, appendicitis, post-surgery (low anterior rectum resection), perforation of pelvic viscera or other causes. In Germany, acute diverticulitis is classified as noncomplicated (German Classification of diverticular disease - CDD type 1a/1b) and complicated diverticulitis (type 2a/b/c). Type 2a and type 2b describe the presence of an abscess (corresponds to Hinchey II classification), whereas the CDD type 2c is associated with free perforation (Hinchey III). 1

Patients suffering from acute complicated diverticulitis benefit from conservative therapy in hospital in case of missing free perforation. In approximately 15% of these patients pelvic abscess will be rising, thus a purely conservative therapy with antibiotics, diet, and analgesia are insufficient to result in complete healing. 2 Instead, abscess drainage is indicated, which in most cases is realized transcutaneously using ultrasound or computed tomography (CT) guidance. If conservative strategies including abscess drainage fail to control a septic event based on abscess, an operative intervention would be preferred. Hartmann's operation or creation of a primary anastomosis with protective ileostomy must be discussed. As early as 1978, the Hinchey working group recommended one of two procedures, depending on the skill of the surgeon and the size of the institution. They recommended either a colostomy with drainage of the perforated left-sided colon, or, in the case of a large institution and sufficient surgical expertise, the primary resection of the diverticula containing section of bowel, with or without anastomosis. 3 And even today, approximately 45 years later, the surgical treatment of complicated diverticulitis with free perforation or sepsis represents a major challenge in clinical practice. Special attention should therefore be paid to improving interventional abscess drainage. The aim must be to control the complicated diverticulitis due to conservative strategies and to bridge the time until surgery into an inflammation-free interval, especially to avoid operations such as a Hartmann procedure. Currently, the methods for drainage of pelvic abscess include CT- or ultrasound- guided percutaneous drainage or surgical drainage. A review published in 2016 examined image-guided percutaneous drainage (pelvic abscess drainage [PAD]) methods. Both ultrasound- and CT-based PAD have proven to be safe and effective widespread techniques with ever-expanding indications. 4 Considering the radiation exposure associated with CT-guided PAD, Azzarello et al named ultrasound-guided PAD as the current gold standard. 5

But what about abscesses which are not accessible for percutaneous drainage? Endoscopic ultrasound-guided PAD (EUS-PAD) is an alternative, minimally invasive tool to drain an abscess, which is localized close to the rectum or left colon, and is therefore not accessible. EUS-PAD was first described by Giovannini et al in 2003. 6 The procedure is performable in a bedside setting, even if the patient is multimorbid or critically ill. In 2021, a systematic review was published, which underlined the effectiveness of EUS-PAD as a minimally invasive treatment option with perfect technical and clinical success and minimal rate of adverse events. 7 Another review was published in 2022, in which the excellent effectiveness and safety of the EUS-PAD was again highlighted. 8

The following cases demonstrate the advantages due to this interdisciplinary strategy with EUS-guided endoluminal PAD in purpose of bridging to surgery.

## First Case

**Fig. 1:**
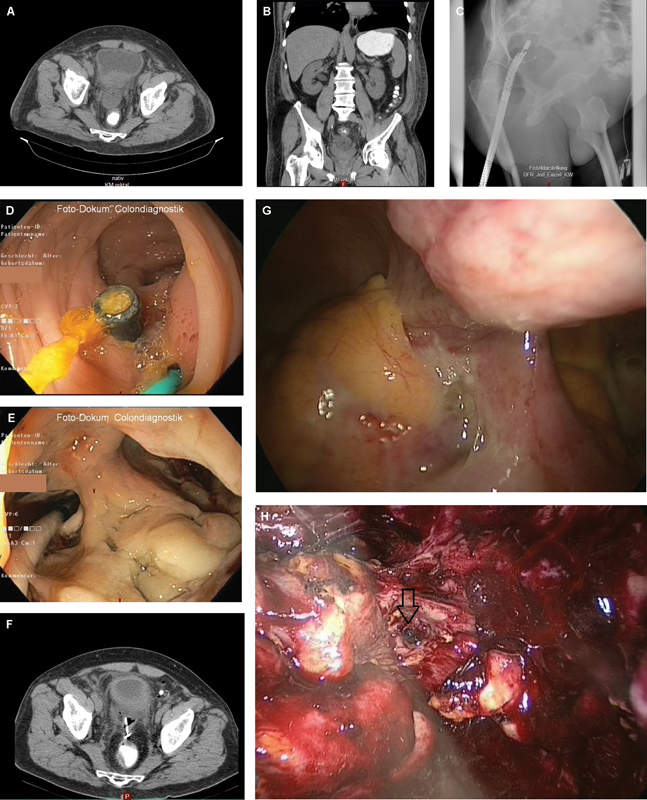
( **A** and **B** ) In a computed tomography (CT) a retrovesical paracolic abscess (10.0 × 4.5 × 6.6 cm) was detected, caused by CDD type 2b (Hinchey II) sigmoid diverticulitis. ( **C** ) X-ray illustration with pigtail stent. ( **D** ) Endoscopy: proper stent position (10 F double pigtail stent, 8 F double pigtail stent 3 cm). ( **E** ) Intubation of abscess after balloon dilatation. ( **F** ) Follow-up CT with decreased abscess with correct stent position. ( **G** ) Intraoperative situs showing pus in the lower abdomen. ( **H** ) Image of the residual abscess with the stent still in place (arrow).

A 62-year-old American Society of Anesthesiologists (ASA) III patient was admitted to hospital with increasing deterioration of general condition, pain in the lower abdomen, and chills. Clinically, there was tenderness in the left lower abdomen and temperature of 38.7°C. Relevant preexisting illnesses include pulmonary artery embolism after thrombosis approximately 9 years ago (long-term medication includes oral anticoagulation: Marcumar), obstructive sleep apnea syndrome under nightly continuous positive airway pressure, and adipositas (body mass index 31 kg/m ^2^ ). Paraclinically, there was a significant inflammatory constellation with C-reactive protein (CRP) value elevation and leukocytosis and the onset of acute kidney failure (CRP 252.8 mg/L, leukocytes 14.40 10^3/μL, creatinine 246 μmol/L). In CT there was a retrovesical paracolic abscess (10.0 × 4.5 × 6.6 cm) detected, caused by CDD type 2b (Hinchey II) sigmoid diverticulitis ( Fig. 1A ). Since there was no possibility of creating an ultrasound- or CT-guided percutaneous abscess drainage, the implantation of an endoluminal drainage at the rectosigmoid junction under endoscopic ultrasound guidance was assessed as feasible. The endoscopist was undertaking flexible endoluminal ultrasound (EUS) with following abscess drainage trans-sigmoidal using two stents (10 F flap stent, 3 cm and 8 F double pigtail stent, 3 cm; Fig. 1B–D ). With additional antibiotic therapy (cefuroxime/metronidazole), the inflammation values and retention parameters declined significantly. A CT and endoscopic follow-up showed a proper stent position and a reduced sized abscess (5.6 × 3 × 2.4 cm) ( Fig. 1E ).

Three weeks later the elective readmission of the patient for resection operation was performed. Preoperative CT showed a regressed abscess with free fluid in the lower abdomen ( Fig. 1F ). Intraoperatively, pus with adherent loops of small bowel was seen ( Fig. 1G ). Laparoscopic anterior rectal/sigmoid resection with a descendorectostomy 9 cm a.a., the excision of residual abscess and stent removal could be performed ( Fig. 1H ). After an unremarkable postoperative course, the patient could be discharged to home on the 10th postoperative day.

## Discussion

The patient suffered from a septic event with renal failure based on a retrovesical abscess due to a complicated sigmoid diverticulitis. With no possibility to create a percutaneous drainage, surgical rehabilitation should be performed according to current guidelines. Because of abscess localization in a retrovesical position, this would most likely have led to a discontinuity sigma resection as Hartmann's procedure, whereby the surgical intervention might also have led to a worsening of the critical clinical situation increasing organ dysfunction. During the trans-sigmoidal drainage phase, an improvement of renal function as well as an end of sepsis therapy could be achieved. Furthermore, the definitive operation can be planned for an interval when the acute infection is under control. There was no need for a protective ileostomy. As a result, the transrectal abscess drainage leads to a reduced risk of perioperative organ dysfunction and need for ileo- or colostomy after surgical resection.

## Second Case

A 52-year-old ASA II patient was admitted to the hospital with left-sided lower abdominal pain for 3 days. Fever, nausea, and vomiting were denied. Clinically, the patient was in a pain-related reduced general condition with local defensive tension in the lower abdomen on the left side. A significant constellation of infection with massively increased infection values was detected in the paraclinical diagnostic (CRP 294 mg/L, leukocytes 12 Gpt/L). Under suspicion of sigmoid diverticulitis, the patient received calculated antibiotic therapy with cefuroxime and metronidazole intravenously. A CT of the abdomen revealed type 2b diverticulitis in the sigmoid colon and colon descendent with covered perforation and retrovesical abscess formation (4.5 × 4.3 × 3.6 cm) in the area of rectosigmoid junction. On the same day a EUS-guided trans-sigmoid abscess drainage (8 F double pigtail stent, 3 cm) was performed. The general condition of the patient subsequently improved rapidly. Complaints and infection levels showed a rapid decrease. A controlling ultrasound showed a significant decrease regarding the size of abscess formation with correct position of the pigtail drainage. Discharge from hospital was possible on the 8th day after admission, whereby early elective operation as laparoscopic sigmoid resection for definitive rehabilitation was advised in the interval. Eight weeks later (postponement of the surgery date by 4 weeks due to a severe respiratory infection) the laparoscopic sigmoid resection with descendorectostomy 12 cm a.a. was performed without need for ileostomy. After an unremarkable postoperative course, the patient could be discharged to home on the 7th postoperative day.

## Discussion

In our case, the patient benefitted from abscess drainage to bridge the time to surgery. Furthermore, the patient had fully recovered from the respiratory infection. Additionally, there was no need for protective ileostomy or a primary colostomy.

The transrectal abscess drainage leads to a reduced risk of perioperative complications due to the anesthesia and to a reduced need for an ileostomy, resulting in significantly lower postoperative morbidity and improved quality of life.

## Conclusion

EUS-PAD is feasible for abscesses that cannot be reached percutaneously in the rectosigmoid junction, thereby the patient can be removed from a septic situation, organ dysfunction, reduction of plasmatic coagulation, or respiratory infection. Apart from the aforementioned advantages of bridging the time to surgery strategy including EUS-PAD, negative side effects of common interventional tools are bypassed. Significantly Lower X-ray exposure, lower costs, shorter duration of hospital stay, and the possibility of bedside intervention, especially in multimorbid patients, are advantages to be mentioned here. 8 In a single-center study, 10 patients underwent transluminal EUS-PAD in diverticular disease with temporary stent placement, published in 2021. 9 Our two cases underline the advantages and effectiveness of EUS-PAD. The Donatelli working group recommends according to their results performing an EUS-PAD in case of abscess size of more than 4 cm and if the abscess is close to the colon wall. 9 The technical and clinical success was achieved in 88.8%. In both of our special cases a laparoscopic operation with primary anastomosis, lower perioperative risk, and without the need of a protective ileostomy in early elective setting became possible by bridging the time to surgery using EUS-PAD.
